# Monopole Antenna with Enhanced Bandwidth and Stable Radiation Patterns Using Metasurface and Cross-Ground Structure

**DOI:** 10.3390/s22218571

**Published:** 2022-11-07

**Authors:** Patrick Danuor, Kyei Anim, Young-Bae Jung

**Affiliations:** 1 Department of Electronics Engineering, Hanbat National University, Daejeon 34158, Korea; 2 Electrical and Computer Engineering, Drexel University, Philadelphia, PA 19104, USA

**Keywords:** cross-ground, frequency-selective surface (FSS), monopole antenna, ocean buoy, omnidirectional, radiation pattern flatness, wideband

## Abstract

In this paper, a printed monopole antenna with stable omnidirectional radiation patterns is presented for applications in ocean buoy and the marine Internet of Things (IoT). The antenna is composed of a rectangular patch, a cross-ground structure, and two frequency-selective surface (FSS) unit cells. The cross-ground structure is incorporated into the antenna design to maintain consistent monopole-like radiation patterns over the antenna’s operating band, and the FSS unit cells are placed at the backside of the antenna to improve the antenna gain aiming at the L-band. In addition, the FSS unit cells exhibit resonance characteristics that, when incorporated with the cross-ground structure, result in a broader impedance bandwidth compared to the conventional monopole antenna. To validate the structure, a prototype is fabricated and measured. Good agreement between the simulated and measured results show that the proposed antenna exhibits an impedance bandwidth of 83.2% from 1.65 to 4 GHz, compared to the conventional printed monopole antenna. The proposed antenna realizes a peak gain of 4.57 dBi and a total efficiency of 97% at 1.8 GHz.

## 1. Introduction

Wireless technology has been one of the fastest growing technologies in the area of communication over the past few years due to the exponential growth in user demand for wireless communication services [1]. Further, Internet-of-Things (IoT) technology has received a lot of focus nowadays due to its wide range of applications, ranging from the human-centric to industry 4.0/5.0 [2,3]. Moreover, the increasing demand for wireless sensor network operations in real-time processing services shifts more attention to IoT-based wireless technologies [4,5]. As IoT devices have stricter requirements for wireless communications specifications, the design of high-efficiency and broadband antenna structures with miniaturized sizes has become one of the significant issues [6,7,8].

**Figure 1:**
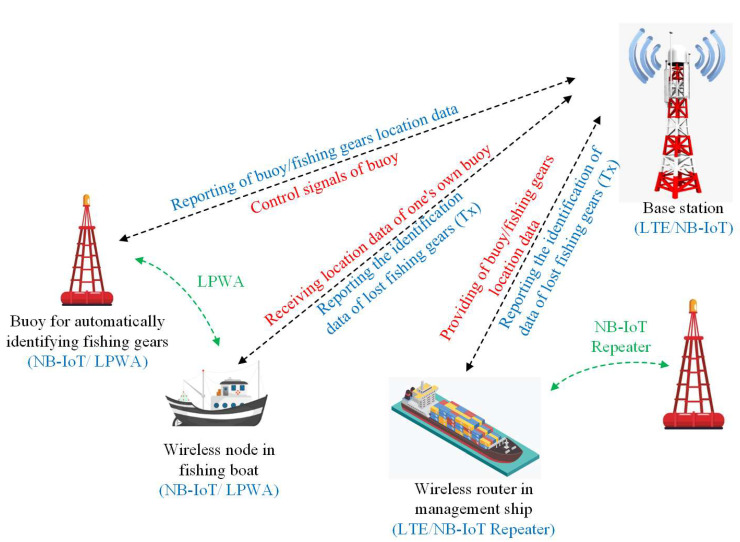
Illustration of marine IoT for fishing gear automatic identification [10].

Marine technology has expanded to include IoT-based wireless devices, and this has paved the way for applications such as ensuring safe ship operations, the safety of ship logistics, etc. [9]. Marine buoy antennas are used to supervise the states of fishing gears in monitoring systems for the real-time systems of electric fishing gears, as illustrated in Figure 1 [10]. The antenna structures for this application require small, lightweight, stable omnidirectional radiation characteristics [10].

Printed monopole antennas are widely used due to the advantages of a low profile, low cost, light weight, ease of fabrication, and integration with other active devices; they can be applied in the fields of radar technology, space science, biomedical research, and wireless communication systems [11,12,13,14,15,16]. Moreover, the advancement of modern wireless systems including marine IoT services also places stringent requirements on printed antennas, such as stable omnidirectional radiation patterns, wide impedance bandwidths, high gain, and reduced design complexity, among others [17]. This has led to various methods being proposed in literature that are aimed at meeting these various design objectives.

A number of techniques have been proposed to enhance the bandwidth performance of conventional printed monopole antennas, which usually have a narrow bandwidth of approximately 2 to 5% [15]. Such methods include increasing the thickness of the substrate, the use of parasitic patches, using substrates with a low dielectric constant, and the use of multi-layer substrates [18,19,20,21,22]. Antenna structures consisting of an electromagnetic bandgap (EBG) coupled with patch antennas have also been proposed to attain surface wave suppression, which results in an improvement of the impedance bandwidth [23,24]. Most of these implementations involve the use of structures that contribute to the complexity of the printed monopole antenna. Further, the use of dielectric resonators (DRs), which are made of low-loss materials with high permittivity, has been one of the proven ways to increase the bandwidth of microstrip antennas [25]. However, this method increases the losses of the antenna, which may degrade its radiation efficiency.

The emergence of metamaterials with different resonator unit shapes has played a great role in reducing the complexity incurred due to the addition of extra structures, thus improving the performance of the antenna [26,27]. Metasurface structures are typically two-dimensional arrays of small scatters or apertures that are geometrically arranged in order to achieve some desirable electromagnetic behavior [28,29,30,31]. Metasurface structures such as frequency selective surfaces (FSSs) have been widely used to improve the low gain and narrow bandwidth performance of planar antennas owing to their low cost and small profile, in addition to their immense transmission and reflection potentials [32,33,34].

The incorporation of FSS structures as superstrate layers to enhance the performance of planar antennas has become a trend nowadays, and it has been investigated by many authors [35]. In [36], a metasurface loaded with a zero-index metamaterial was used as a superstrate to enhance the gain of a patch antenna. However, this method tends to result in relatively complex structures with large electrical sizes.

To achieve more simplified structures, antennas with metamaterial loading near or around the patch have been proposed. In [37,38], antenna structures that achieved a wide impedance bandwidth performance using metamaterials and metasurface structures were proposed. In addition, in [39], an antenna combined with a fractal metasurface was proposed, which led to the enhancement of the impedance bandwidth. Moreover, in [40], a monopole antenna loaded with a single negative (SNG) metamaterial was proposed for ultra-wideband (UWB) applications. Even though these antennas exhibit good wideband and gain performances, they suffer from distorted omnidirectional radiation patterns at various frequencies over their operating bandwidths.

To attain stable omnidirectional radiation patterns over the entire operating frequency band, the authors in [41] proposed a conductor ring and a circular slot with a quarter-wavelength width placed around a patch antenna to suppress the side radiations and stabilize radiation patterns over a large bandwidth. Moreover, in [42], the authors employed two dipoles in a parallel configuration over a reflector to achieve stable radiation patterns and a nearly identical E- and H-plane. Furthermore, a differential feed is presented in [43] as a solution to achieving more stable and omnidirectional radiation pattern characteristics of a single-fed circular monopole antenna over its operating frequency range. However, these radiation-pattern stability techniques resulted in relatively complex structures. A simplified wideband antenna structure with stable omnidirectional radiation patterns is proposed in [44]; however, a relatively lower gain was recorded in the operating frequency band.

A cross-plate monopole antenna formed by two metal plates having a 3D-like geometry configuration was proposed in [45,46] to achieve a wide impedance bandwidth and improved radiation pattern characteristics. The antenna structure in [45] is formed by two orthogonal step-shaped metal plates, and in [46], mutually crossed metal fins serving as a radiator are utilized. These structures, however, have high complexity and may suffer from poor co-to-cross polarization.

In this paper, a relatively simple printed monopole antenna structure with stable omnidirectional radiation patterns is designed with two FSS unit cells and a cross-ground structure. The FSS unit cells are placed at the backside of the antenna, which maintains the planar form of the antenna. The cross-ground structure is implemented to achieve stable monopole-like radiation patterns over the entire operating band of the antenna. Meanwhile, the FSS unit cells exhibit resonant characteristics, which improves the impedance bandwidth of the antenna. A peak gain of 4.57 dBi and an impedance bandwidth of 83.2% is realized. Moreover, the antenna achieves a total efficiency of 97%. The proposed antenna is designed for the applications of ocean buoy in the marine IoT for the automatic identification of fishing gears.

## 2. Antenna Geometry and Configuration

### 2.1. Conventional Monopole Antenna

**Figure 2:**
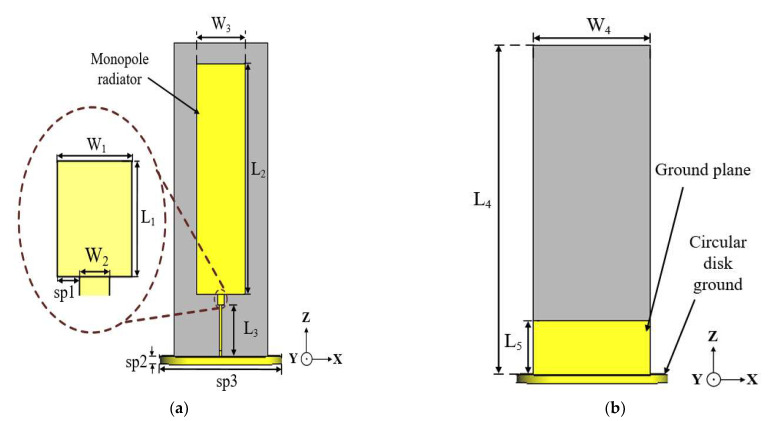
Geometry of the conventional monopole antenna: (**a**) front view and (**b**) back view.

The conventional monopole antenna, as already established, usually suffers from a narrow impedance bandwidth and low gain. Moreover, for applications in ocean buoy, a stringent requirement of stable radiation patterns is desired to achieve the target performance [10]. The geometry of the conventional monopole antenna is shown in Figure 2.

The antenna is composed of a microstrip-fed rectangular radiator realized on a Taconic TLY-5 substrate with a relative permittivity ($\varepsilon_{r}$) of 2.2, a thickness (*h*) of 0.508 mm, and a loss tangent (tan δ) of 0.0009.

The antenna has dimensions of 43 mm × 121.5 mm (0.38 $\lambda_{0}$ × 0.73 $\lambda_{0}$), where $\lambda_{0}$ represents the free-space wavelength at the design frequency of 1.8 GHz. The length of the radiator is designed at 34λg, ($\lambda_{g}$ corresponds to the guided wavelength at 1.8 GHz) to excite higher modes of the monopole antenna to achieve the tilted radiation pattern beams necessary for the ocean buoy application. The antenna is fed by a 50-Ω SMA connector and situated on a circular disk ground with a diameter of 28 mm. A stub with the length L_1_ is placed between the radiator and the feedline to obtain good impedance matching.

The conventional monopole antenna has an impedance bandwidth of 16.2% ranging from 1.7 to 2 GHz, with an average gain of 3.7 dBi at the operating frequency band.

### 2.2. Conventional Antenna Incorporated with Cross-Ground

**Figure 3:**
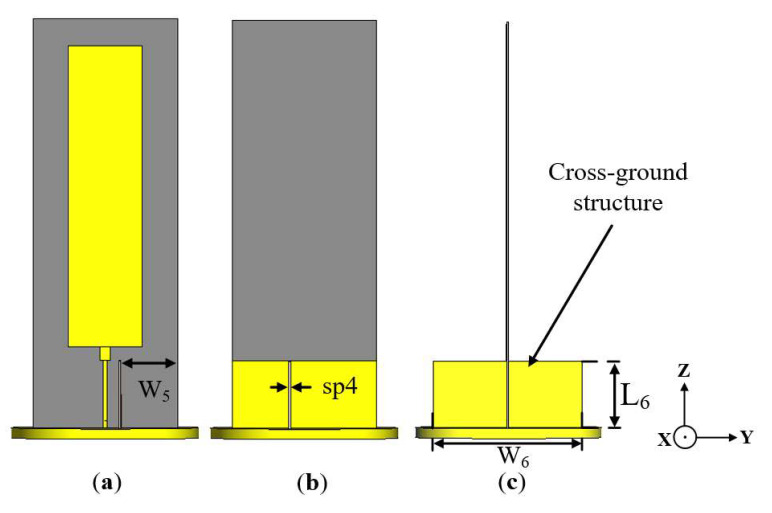
Geometry of the conventional antenna incorporated with cross-ground structure: (**a**) front view, (**b**) back view, and (**c**) side view.

**Figure 4:**
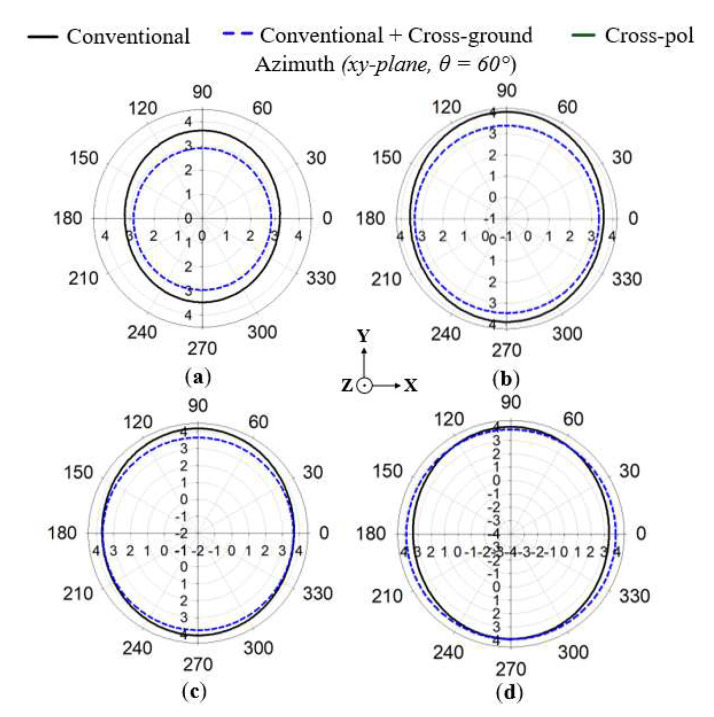
Simulated results of the azimuth radiation patterns for (**a**) 1.7, (**b**) 1.8, (**c**) 1.86, and (**d**) 2 GHz.

In order to achieve stable omnidirectional radiation patterns throughout the antenna’s operating band, a cross-ground structure is incorporated into the antenna design, as shown in Figure 3. This is achieved by aligning a rectangular substrate, with copper printed on both sides, perpendicular to the printed ground plane at the backside of the antenna. The cross-ground is situated on the circular ground plane, which serves as the base and support to the antenna structure. The cross-ground acts as a reflector to maintain omnidirectional radiation patterns with respect to the azimuth (*xy*-plane). The simulated radiation patterns at 1.7, 1.8, 1.86, and 2 GHz are shown in Figure 4a–d, respectively. The azimuth radiation patterns (*xy*-plane) are measured at a theta (*θ*) cut angle of 60° (i.e., in the direction of the main radiation lobe).

From the results, it is evident that consistent omnidirectional radiation patterns are maintained within the operating band of the antenna (i.e., 1.7–2 GHz) when the cross-ground structure is added. However, with the incorporation of the cross-ground, the gain is seen to depreciate slightly, especially for lower frequencies of the operating band, as depicted in Figure 4.

**Figure 5:**
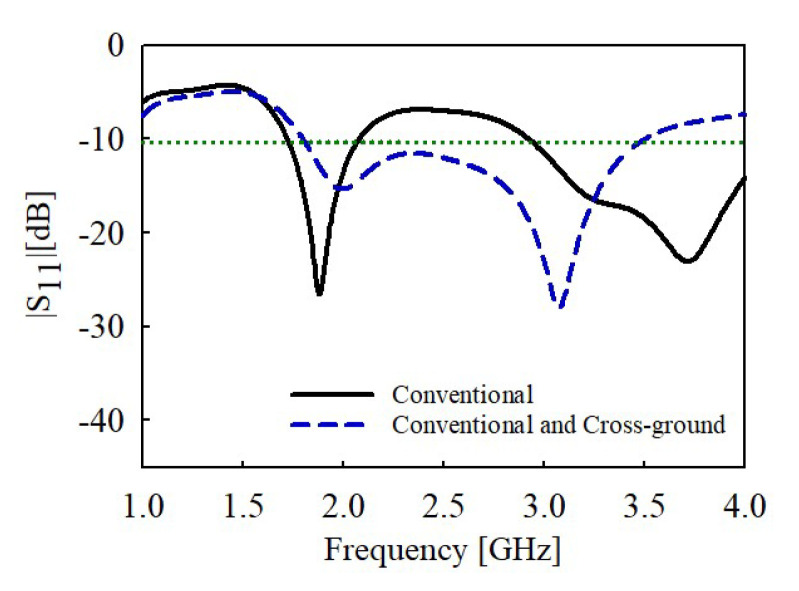
Simulated reflection coefficient of the conventional monopole only and with cross-ground structure.

Further, with the addition of the cross-ground structure, the impedance bandwidth is broadened for |S_11_| < −10 dB from 1.8 to 3.5 GHz, as shown in Figure 5. This is due to the improvement of the matching conditions augmented by the addition of the cross-ground to the antenna design.

### 2.3. Monopole Antenna Incorporated with Both Cross-Ground Structure and FSS Unit Cells

**Figure 6:**
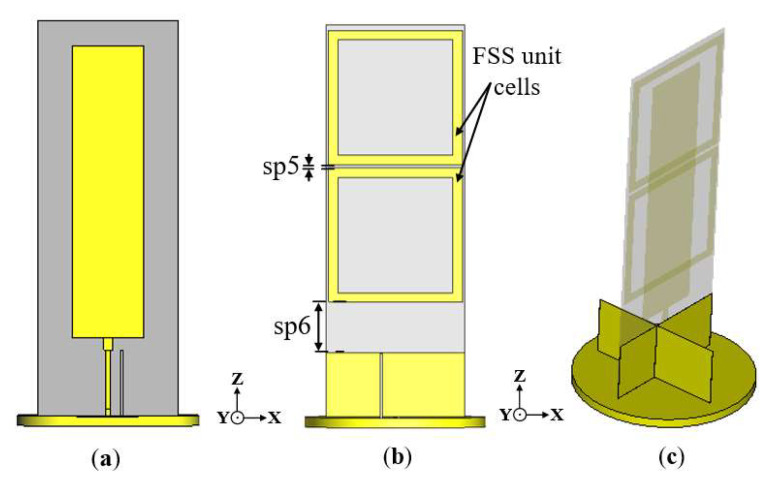
Geometry of proposed antenna with both cross-ground structure and FSS unit cells: (**a**) front view, (**b**) back view showing FSS unit cells, and (**c**) side view.

To improve the gain of the conventional monopole antenna incorporated with the cross-ground targeting the lower frequencies of the operating band, two frequency-selective surface (FSS) unit cells were designed and incorporated into the backside of the antenna, as shown in Figure 6. FSS structures are metasurfaces that merely exhibit an electric response. FSSs can modify the incident electromagnetic wave to attain reflectivity or transmissivity [33]. FSS structures exhibit dispersive and angular stability properties that can be exploited to achieve high gain characteristics [34]. Moreover, the element shape, size, and periodicity of the FSS unit cell structure can result in resonances.

The FSS unit cell is designed with dimensions to satisfy the spatial reflective characteristics at the resonance frequency of 1.8 GHz. The dimensions of the FSS unit cells are obtained through parametric optimizations.

**Figure 7:**
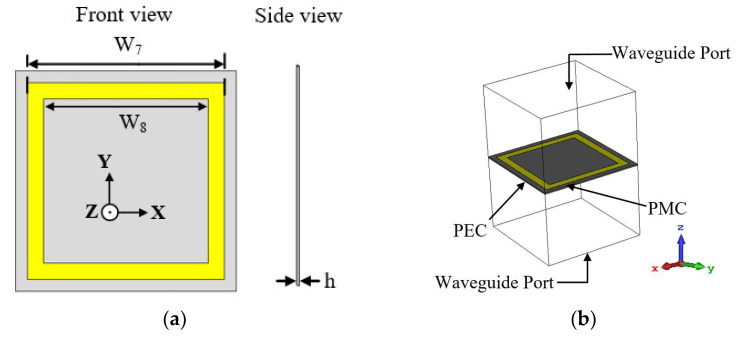
Geometry of proposed FSS unit cell: (**a**) 2D view and (**b**) 3D view of simulation setup.

The geometry of the proposed FSS unit cell structure is illustrated in Figure 7a. It is composed of a metallic square ring of size 41 mm × 41 mm (0.37 $\lambda_{g}$ × 0.37 $\lambda_{g}$) that resides on a Taconic TLY-5 substrate. The boundary conditions of the simulated model of the FSS unit cell are illustrated in Figure 7b. The unit cell is simulated over 1 to 2 GHz, with the structure placed between two waveguide ports, which are situated on each side of the *z*-axis.

**Figure 8:**
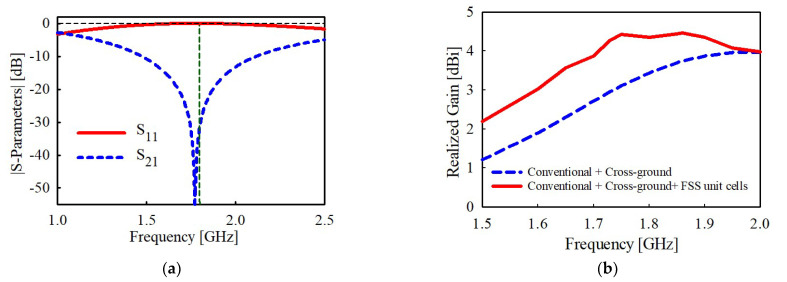
Simulation results of (**a**) the reflection and transmission coefficient (S_11_ and S_21_, respectively) of the proposed FSS unit cell and (**b**) gain results comparison after incorporation with FSS unit cells.

The transmission and reflection coefficient results of the FSS unit cell are shown in Figure 8a. The results demonstrates that the FSS unit cell exhibits reflective characteristics at the target frequency of 1.8 GHz.

In Figure 8b, the 3D peak realized gain of the monopole antenna is shown, where a gain increment of about 1 dBi is realized within the lower operating band of the antenna (i.e., 1.7, 1.8, and 1.86 GHz). The gain, however, depreciates for higher frequencies due to the narrow band properties of FSS structures [34]. The optimized dimensions of the proposed antenna and the FSS unit cell structure are outlined in Table 1.

**Figure 9:**
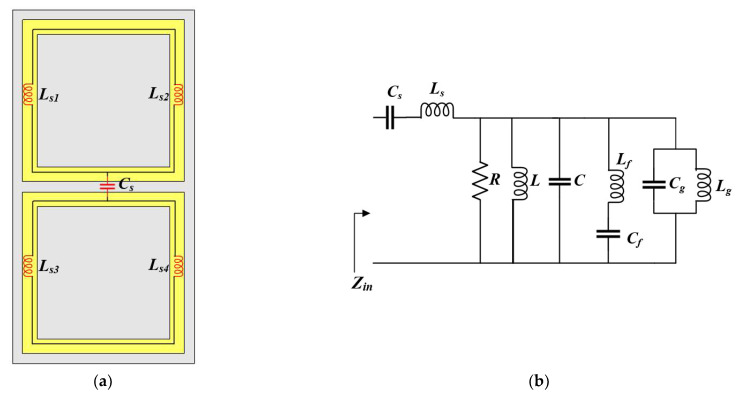
Illustration of (**a**) FSS unit cell layer with inductive and capacitive components and (**b**) equivalent circuit model of proposed antenna structure with both cross-ground and FSS unit cells.

The proposed antenna structure exhibits a manner of coupling between the FSS unit cells. The FSS unit cell layer, which is comprised of metallic square loops, can be modeled as a combination of inductance (*L_f_*) and capacitance (*C_f_*), as illustrated in Figure 9a. The equivalent circuit of the proposed antenna incorporated with both FSS unit cells and the cross-ground structure is given in Figure 9b, where (*Z_in_*) represents the input impedance of the antenna.

**Figure 10:**
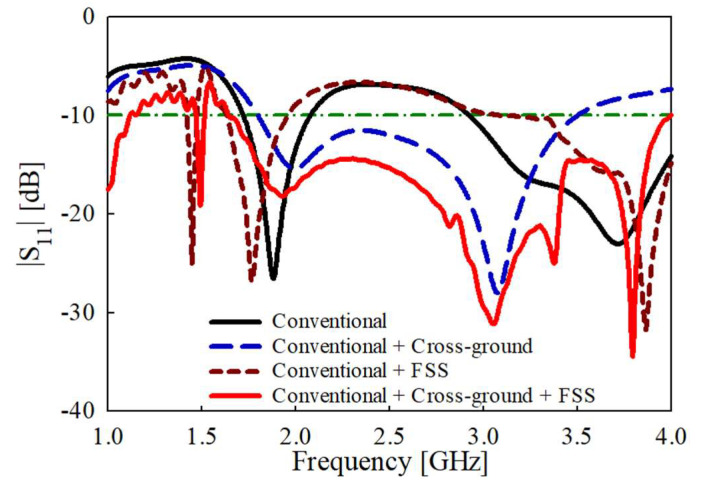
Simulated results of the input reflection coefficient amplitude.

The matching stub capacitance and inductance are represented by (*C_s_*) and (*L_s_*), respectively. The circuit theory concept has been utilized to analyze the bandwidth characteristics of the antenna. The cross-ground structure can also be modelled as a combination of inductance (*L_g_*) and capacitance (*C_g_*). This gives rise to more capacitive and inductive components that enhance the matching conditions to achieve a wider impedance bandwidth of 83.2% from 1.65 to 4 GHz, as shown in Figure 10.

**Figure 11:**
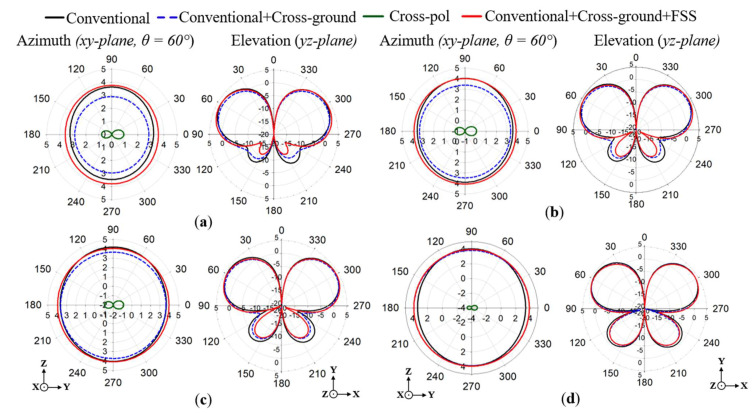
Simulated results of the azimuth and elevation radiation patterns for (**a**) 1.7, (**b**) 1.8, (**c**) 1.86, and (**d**) 2 GHz.

The results of the simulated azimuth (*xy*-plane) and elevation (*yz*-plane) radiation patterns are given in Figure 11a–d at 1.7, 1.8, 1.86, and 2 GHz, respectively. The radiation patterns are compared between the proposed antenna (i.e., both cross-ground and FSS added) and the conventional antenna with and without the cross-ground structure. The elevation radiation patterns were recorded for the *yz*-plane of the monopole antenna structure. It can be observed that the direction of the main radiation lobe corresponds to an angle of about 60°. This is as a result of the target length of the monopole antenna designed at 34λg to generate a tilted beam direction. In the radiation pattern results, a gain of more than 3.5 dBi was realized for all the frequencies. Meanwhile, lower side lobe levels were recorded for the proposed monopole antenna as compared to the conventional one. The azimuth radiation patterns (i.e., which correspond to the *xy*-plane of the monopole antenna structure) are recorded at a cut angle of 60° with respect to the main lobe direction of the elevation plane.

From the results in Figure 11, it can be realized that for the proposed monopole antenna, stable omnidirectional radiation patterns are maintained for all the frequencies, unlike that of the conventional monopole antenna. The cross-polar components of the proposed monopole antenna are plotted in Figure 11. It can be observed that low cross-polar levels are achieved for all radiation patterns at the various frequencies.

## 3. Measurement Results and Discussion

**Figure 12:**
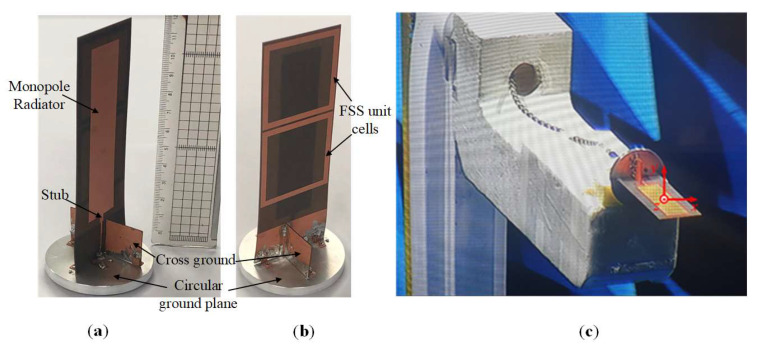
Photograph of the fabricated monopole antenna: (**a**) front view, (**b**) back view, and (**c**) far-field measurement set-up.

To validate the proposed antenna, a prototype was designed and fabricated, as shown in Figure 12a,b. The reflection coefficient of the fabricated antenna is measured by means of a vector network analyzer (VNA), and the gain and radiation patterns are verified in the far-field anechoic chamber, as displayed in Figure 12c.

**Figure 13:**
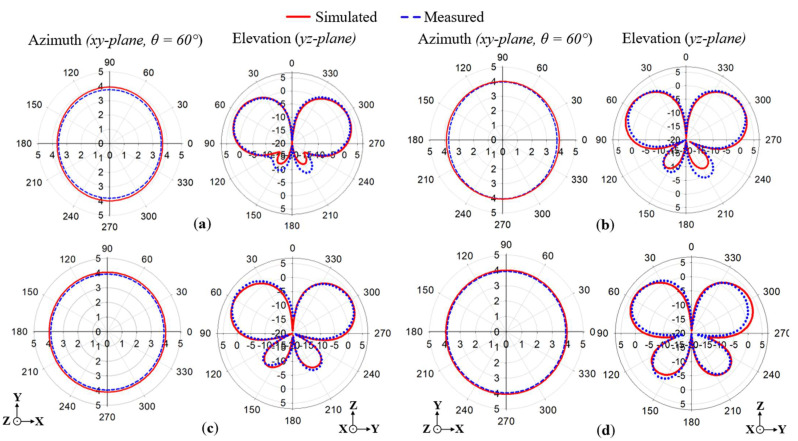
Simulated and measured results of the azimuth and elevation radiation patterns for (**a**) 1.7 GHz, (**b**) 1.8 GHz, (**c**) 1.86 GHz, and (**d**) 2 GHz.

Figure 13 presents the simulated and measured radiation patterns of the proposed antenna in the azimuth and elevation (*xy* and *yz*-planes, respectively) at 1.7, 1.8, 1.86, and 2 GHz. The simulated and measured results are in excellent agreement, which validates that the proposed monopole antenna exhibits flat omnidirectional radiation patterns across the antenna’s operating band.

**Figure 14:**
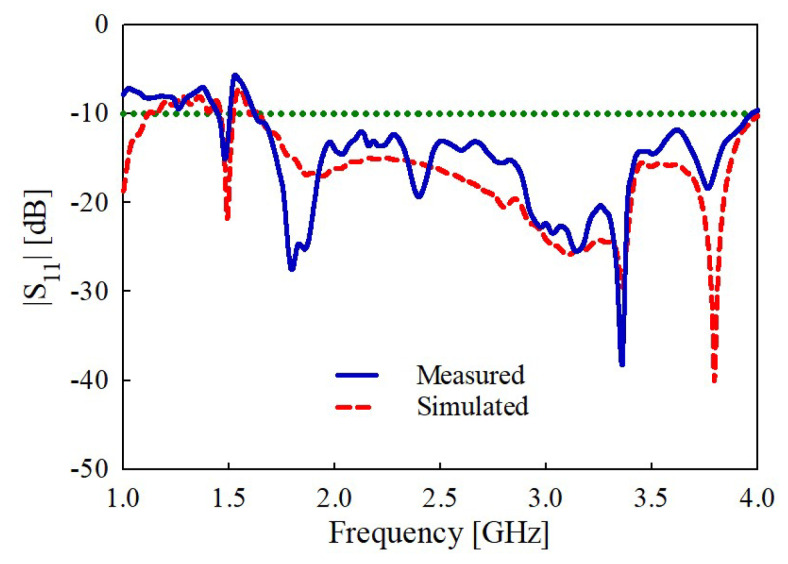
Simulated and measured results of the input reflection coefficient amplitude.

The simulated and measured |S_11_|results of the proposed antenna are shown in Figure 14. It can be observed that the simulated results agree well with the measured ones. However, the slight discrepancy between the measured and simulated results at the frequency points of 1.8, 3.3, and 3.8 GHz is due to the imperfect contact of the cross-ground plane with the circular-disk plane during fabrication. Regardless, both the simulated and measured results verify that the antenna offers a wide impedance bandwidth (|S_11_| < −10 dB) of 83.2% ranging from 1.65 to 4 GHz.

**Figure 15:**
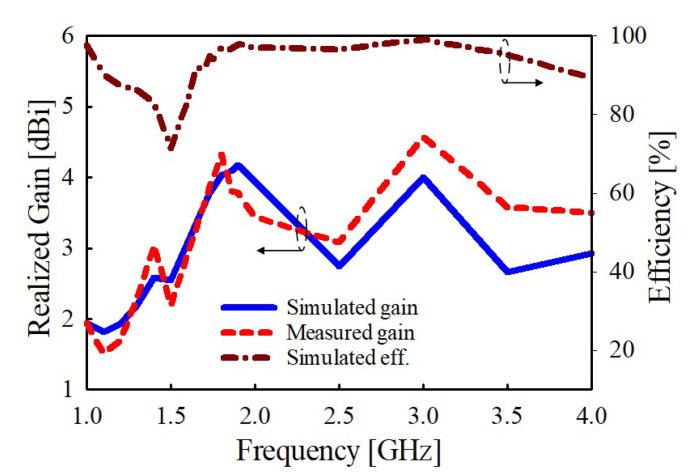
Results of the simulated and measured gain and antenna efficiency.

Moreover, the simulated and measured gains of the proposed antenna are presented in Figure 15. The antenna gain varies from about 3.2 to 4.57 dBi, with a peak gain of 4.57 dBi recorded at 1.8 GHz. The discrepancies in the simulated and measured gain results in the higher frequencies may be attributed to measurement error. Likewise, a plot of the total efficiency with frequency variation is shown in Figure 15, which shows that the proposed antenna achieves a maximum efficiency of about 97% at 1.8 GHz.

To further highlight the superiority of the proposed antenna, a comparative analysis between the proposed antenna and other reported antenna structures is presented in Table 2, summarizing the antenna’s characteristics in terms of impedance bandwidth, 3-dB gain bandwidth, peak gain, dimensions, and maximum efficiency. It can be inferred from Table 2 that the proposed antenna realizes a comparatively better impedance bandwidth than the other antennas, with the exception of reference [5,6,7]. However, the proposed antenna has a relatively higher gain than the antenna structures proposed in [5,6,7]. Further, compared to the other antennas, the proposed antenna has a relatively higher efficiency and the added advantage of stable omnidirectional radiation patterns, which is necessary for ocean buoy and marine IoT applications.

## 4. Conclusions

A printed monopole antenna with stable omnidirectional radiation patterns has been presented in this paper. The antenna integrates a layer of FSS consisting of two unit cells and a cross-ground structure into the antenna design. The proposed antenna attains stable monopole-like omnidirectional radiation patterns throughout the operating band. Furthermore, the incorporation of both the FSS and cross-ground structure resulted in a wide impedance bandwidth of 83.2% from 1.65 to 4 GHz. A high efficiency and peak gain of 97% and 4.57 dBi, respectively, was achieved, making the proposed antenna suitable for applications in ocean buoy and the marine IoT.

## Figures and Tables

**Table 1:** Geometrical parameters of the proposed antenna.

Parameter	Value (mm)	Parameter	Value (mm)
L_1_	4	W_5_	16.9
L_2_	89.5	W_6_	44.5
L_3_	20	W_7_	41
L_4_	121.5	W_8_	35
L_5_	20	sp1	1
L_6_	20	sp2	3
W_1_	3	sp3	56
W_2_	1.2	sp4	0.5
W_3_	22	sp5	0.6
W_4_	43	sp6	15.6

**Table 2:** Comparison between the proposed antenna and existing antenna structures.

Reference	* Impedance Bandwidth	3-dB Gain Bandwidth	Peak Gain (dBi)	Size (L × W)	Maximum Efficiency
[11]	1.9–2.33 GHz (20.33%)	3.06%	3.35	40 × 100 mm^2^	66%
[12]	0.47–1.21 GHz (87.54%)	-	1.19	40 × 231 mm^2^	92.4%
[13]	1.07–3.36 GHz (103.39%)	27.2%	3.7	40 × 115 mm^2^	-
[14]	0.78–2.25 (97%)	-	3	55 × 30 mm^2^	-
[38]	1.82–1.98 GHz (8%)	-	4.3	86.5 × 86.5 mm^2^	98%
[39]	2.3–4 GHz (53.97%)	-	3.6	40 × 40 mm^2^	90%
[47]	2–3.8 GHz (62%)	-	3.2	40 × 40 mm^2^	90%
Conventional	1.7–2 GHz (16.2%)	30%	3.7	40 × 121.5 mm^2^	91.5%
Proposed	1.65–4 GHz (83.2%)	37.03%	4.45	43 × 121.5 mm^2^	97%

* The bandwidth of |S_11_|< −10 dB.

## Data Availability

Not applicable.
